# Liposomes-in-Gel as the Docetaxel Delivery for the Effective Treatment of Psoriasis by Inhibiting the Proliferation of Blood Vessels

**DOI:** 10.3390/gels11040228

**Published:** 2025-03-22

**Authors:** Ruoyang Jia, Yinyin Liu, Yifang Wu, Si Shen, Keang Cao, Xue Chen, Yang Wu, Wang Shen, Lu Wang, Bin Sun, Yongli Zhang, Hongmei Xia

**Affiliations:** College of Pharmacy, Anhui University of Chinese Medicine, Hefei 230012, China; 2022205221003@stu.ahtcm.edu.cn (R.J.); lyy123@stu.ahtcm.edu.cn (Y.L.); wuyifang@stu.ahtcm.edu.cn (Y.W.); shensi@stu.ahtcm.edu.cn (S.S.); 2023205221003@stu.ahtcm.edu.cn (K.C.); 2023205221004@stu.ahtcm.edu.cn (X.C.); 2023205227034@stu.ahtcm.edu.cn (Y.W.); 15155034624@163.com (W.S.); 15155063158@163.com (L.W.); 13721231091@163.com (B.S.); 18119742560@163.com (Y.Z.)

**Keywords:** liposomes-in-gel delivery system, docetaxel, psoriasis, IL6-HIF-1α-VEGF axis

## Abstract

Psoriasis is a chronic skin disease caused by the interaction of multiple factors that leads to the abnormal growth of stratum corneum cells and has been called an immortal cancer. Docetaxel has been trialed for the treatment of psoriasis due to its superior ability to induce apoptosis, but its insolubility and low bioavailability have hampered its development. Here, docetaxel (DTX)-loaded liposomes-in-gel (DTX-LP-G) as the transdermal delivery was investigated to the treatment of psoriasis via modulating the IL6-HIF-1α-VEGF axis. The results demonstrated that DTX-LP-G cumulatively released a much higher amount of drug into the skin than that from DTX-loaded liposomes (DTX-LPs) and DTX-loaded gel (DTX-G). DTX-LP-G was also the most efficient in scavenging hydrogen peroxide free radicals in vitro. In a mouse model of psoriasis, DTX-LP-G acted as a preliminary therapeutic agent for psoriasis in terms of apparent evaluation, splenomegaly, suppression of MDA content in skin tissue, and down-regulated the expression of IL6, HIF-1α, and VEGF to control the proliferation of vessels, except for a less pronounced effect on the stratum corneum. In addition, enrichment analysis can speculate that DTX also treated psoriasis by resisting the production of keratin-forming cells.

## 1. Introduction

Psoriasis is a chronic skin disease that is caused by the interaction of multiple factors, resulting in the appearance of scaly erythema on the skin [1]. So far, the literature reports have shown that psoriasis was due to a disorder of the immune system, resulting in skin inflammation because of the significant release of cytokines by T cells [2,3,4]. In addition, the growth cycle of keratinocytes becomes shorter, leading to the accumulation of a large number of dead cells in the stratum corneum, and the appearance of symptoms like dandruff [5]. Therefore, the traditional treatment methods aim to slow down or make the disappearance of skin surface erythema and restore the normal growth cycle of keratinocytes [6]. However, in recently reported studies, it was identified that psoriasis could be effectively treated and prevented by removing reactive oxygen species (ROS), which also indicated the involvement of ROS in the mechanism of psoriasis [7,8]. The accumulation of excess ROS in skin cells creates a state of oxidative stress that stimulates the release of large amounts of inflammatory factors from various cells such as neutrophils, macrophages, and T helper cells (Th) [9]. Therefore, inhibiting the expression of inflammatory factors and restoring the redox balance by modulating the inflammatory signaling pathway is a new idea for alleviating the symptoms of psoriasis.

Taxanes can control the depolymerization of β-tubulin, stop cell division, and lead to cell death [10]. Therefore, Yin et al. developed an ointment without water phase to treat psoriasis by inducing the apoptosis of stratum corneum cells with abnormal hyperplasia [11]. In addition, the encapsulation of paclitaxel into nanospheres modified by tyrosine not only proved that nanocarriers could enhance the effective delivery of the hydrophobic paclitaxel across the skin to the whole body, but also verified that transdermal drug delivery could serve as a potential therapy for psoriasis [12]. Docetaxel (DTX), a second-generation synthetic compound, is superior to paclitaxel in terms of hydrophilicity and pharmacologic activity [13]. It demonstrated DTX had antioxidant activity in our previously reported article. Moreover, the association between DTX and psoriasis was further searched. Therefore, a novel, safe and effective vehicle delivered DTX across the skin was designed, for targeted treatment of psoriasis.

Psoriasis treatments primarily include corticosteroids and biologics like TNF-α inhibitors. While corticosteroids are effective, their long-term use can cause side effects such as skin thinning. Biologics offer strong efficacy but are expensive, require injections, and may suppress the immune system. DTX-LP-G, a novel formulation, offers a promising alternative by targeting the affected areas more precisely, reducing side effects and cost. The liposomes-in-gel formulation of DTX-LP-G provides additional benefits over conventional treatments. Liposomes enhance drug stability and targeted delivery, while the gel improves skin adherence, controlled release, and patient compliance. Together, these features make DTX-LP-G a safer, more effective, and convenient treatment for psoriasis.

Liposomes-in-gel is a new type of formulation providing transdermal drug delivery, in which liposomes with a lipid bilayer as a double drug release barrier are dispersed in a natural polymer gel, forming a three-dimensional network structure of the gel [14,15]. The use of liposomes with phospholipids and cholesterol (CHO) as the main materials for forming a lipid membrane structure has been reported, its safety was beyond doubt [16], and the lipid membrane structure is similar to the cell membrane, which could effectively deliver drugs into the cells [17,18]. In the liposomes-in-gel system, polysaccharides are commonly used to form three-dimensional network structure and improve stability, such as hyaluronic acid [19], chitosan [20], sodium alginate [21], and other materials. Among them, hyaluronic acid (HA), as a natural, non-immunogenic polysaccharide, interacts with CD44 and hyaluronan-mediated motility receptor (HAMM) to regulate inflammatory and carcinogenic activities [22]. In addition, the combination of HA with liposomes as the drug delivery system enhances drug penetration into the stratum corneum. Hyaluronic acid-modified novel mutable liposomes target the CD44 receptor on the surface of psoriatic cells and inhibit the secretion of inflammatory factors [23]. Therefore, a gel based on the HA matrix can exert targeting and anti-inflammatory effects for the treatment of psoriasis.

In this study, a liposomes-in-gel system as DTX delivery was characterized in relation to its physical properties, their release behavior was studied in vitro and ex vivo, including the inhibitory activity of hydroxyl radicals. The focus was on whether DTX and DTX-loaded formulations could inhibit the abnormal division of stratum corneum cells to achieve the purpose of treating psoriasis, the model of psoriasis-like mice induced by imiquimod (IMQ) was established, and also the therapeutic effects were analyzed from multiple perspectives.

## 2. Results and Discussion

### 2.1. Prescription and Characterization of DTX-Loaded Formulations

**Figure 1:**
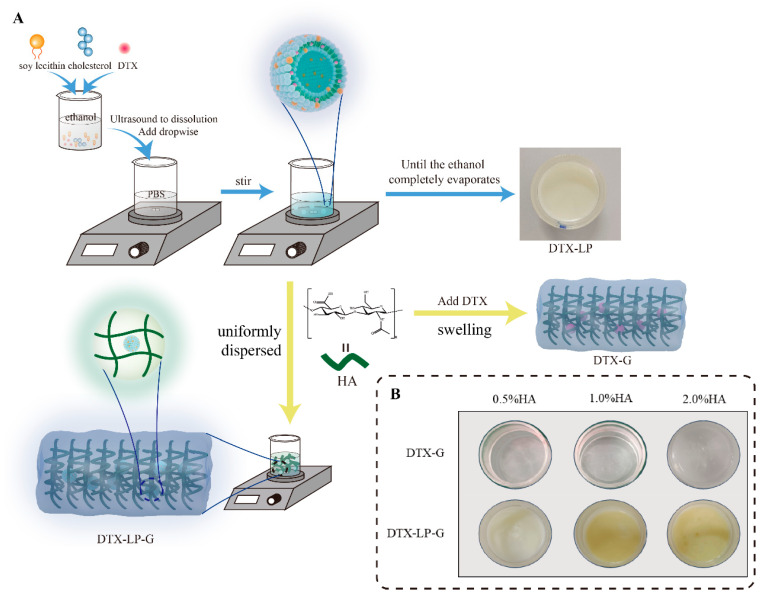
Preparation process and characterization of DTX-loaded formulations. (**A**) DTX-LP was prepared by injection method, and then HA was evenly sprinkled into the liposome suspension to obtain DTX-LP-G. DTX was added to HA and naturally swelling to obtain DTX-G. (**B**) Appearance of DTX-G and DTX-LP-G with the different concentration of HA.

DTX-LP-G was prepared in two steps, the first step to construct the liposome structure and the second step to disperse the liposome uniformly into the hyaluronic acid hydrogel (Figure 1A). The ratio of CHO:SL was crucial for the effect of liposome physicochemical properties [24]. Referring to previous reports on the optimization of liposome preparation, we chose cholesterol and soy lecithin (SL) ratios of 1:3, 1:5, and 1:10 to prepare drug-containing liposomes and characterized them. As shown in Figure 1B and Table 1, it was observed under cryo-electron microscopy that DTX-LP was spheroidal and uniformly distributed. Only when the ratio of CHO:SL was 1:10, the prepared DTX-LP showed vesicle deformation and aggregation, which might be related to the increased amount of soy lecithin. Then, the liposomes prepared with three ratios were negatively charged and the pH was close to 7.4. When CHO:SL was 1:5, the particle size was the smallest (143.37 ± 2.04 nm), the encapsulation efficiency (EE%) was the highest (88.50 ± 1.45%), the drug loading rate (DLR%) was 0.734 ± 0.012%, and the potential was −36.08 ± 1.08 mV. Therefore, the optimal ratio of CHO:SL for DTX-LP was 1:5.

Next, the appearance of DTX-G prepared with HA as the gel matrix was white (Figure 1B), and with the HA concentration increasing, the viscosity increased significantly, and the fluidity decreased [25]. When the HA concentration was 0.5%, the prepared DTX-G was too liquid to adhere to the skin surface, which could easily cause drug loss. In addition, the pH value of DTX-G was between 7.26 and 7.27. When DTX-G contained 1.0% HA, the drug loading rate was significantly higher than that of DTX-G prepared with 2.0% HA (Table 2). In addition, HA was combined with the liposome delivery system to construct a liposomes-in-gel delivery system, which provided good biocompatibility. When the HA concentration was 0.5%, DTX-LP-G was milky white in color. When the HA concentration was 1.0% and 2.0%, DTX-LP-G was light yellow (Figure 1B). The pH value of DTX-LP-G was around 7.4, which was closer to the pH value of body fluids. Moreover, the increase in viscosity improved the adhesion of DTX-LP-G to the skin but also made it difficult to evenly spread DTX-LP-G prepared with 2.0% HA. Therefore, DTX-LP-G prepared at a concentration of 1.0% HA was optimal.

### 2.2. In Vitro Drug Release of DTX-Loaded Formulations

**Figure 2:**
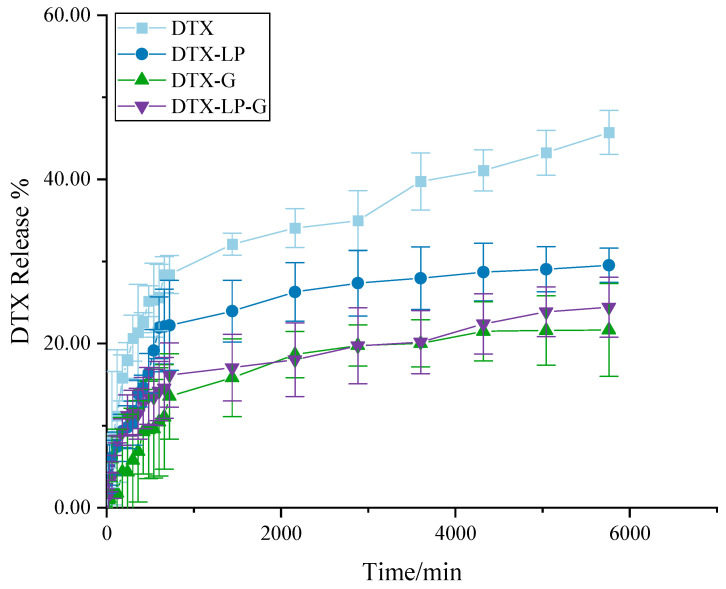
The results of in vitro drug release.

Drug release and retention were crucial for the evaluation of topical formulations. Therefore, in vitro release experiments were performed to explore the in vitro release pattern of DTX-loaded formulations, and the results are shown in Figure 2. Firstly, both DTX and its formulations showed a stable and slow-release effect over 96 h. Among them, DTX sustained the highest cumulative drug release rate of 45.73 ± 2.68% at 96 h. This was followed by the DTX-LP and then DTX-G. Finally, DTX-LP-G had the lowest cumulative drug release rate. This might be due to the fact that DTX was constrained by the three-dimensional network structure of hydrogel and blocked by the phospholipid bilayer, and it might be due to the stabilizing interacting forces formed between DTX and HA, cholesterol, or soy lecithin.

### 2.3. Skin Permeation Study of DTX-Loaded Formulations

**Figure 3:**
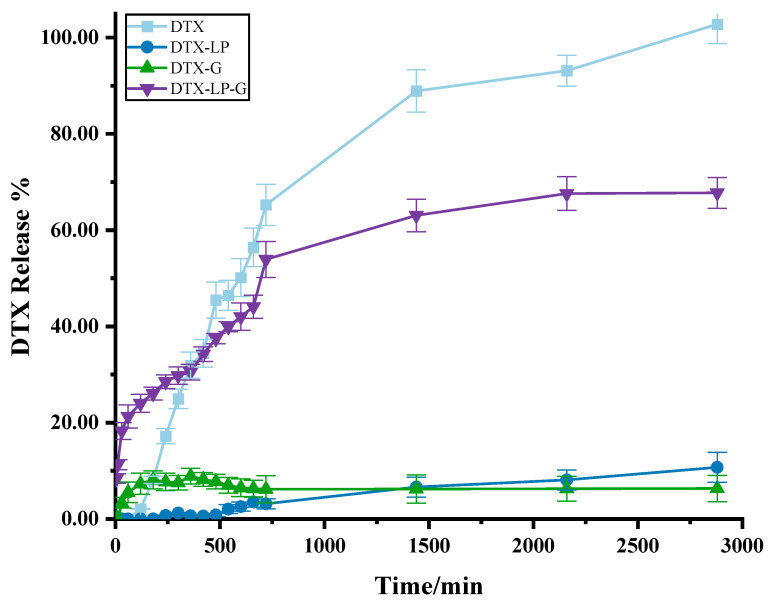
The results of ex vivo skin penetration.

Since DTX-LP-G showed the lowest release rate compared to DTX-LP and DTX-G in the release assay in vitro, the suitability of DTX-LP-G was further determined for dermal administration by the penetration rate of the drug in the isolated skin. Unexpectedly, in the skin permeation test ex vivo, the cumulative amount released from the DTX solution exceeded 80% within 24 h (Figure 3), which was higher than that of DTX-LP, DTX-G, and DTX-LP-G, suggesting that all the three dosage forms including liposomes, gel, and liposomes-in-gel had the function of the slow release of the drug and prolonging the duration of the drug’s action [26]. Among the DTX-loaded formulations, DTX-LP-G significantly penetrated into the skin at a significantly higher amount than that from DTX-LP and DTX-G. Within the same time frame, the cumulative release of DTX-LP-G reached 67.64 ± 3.49%, whereas the cumulative release of DTX-LP and DTX-G was less than 20%. This result strongly demonstrates that DTX-LP-G significantly enhances skin permeability.

DTX-LP-G typically offer better stability due to the protection provided by the gel matrix, preventing aggregation and settling of liposomes. In contrast, nanoemulsions may experience phase separation or aggregation over time [27]. Solid lipid nanoparticles (SLNs) are generally more stable and less prone to crystallization, offering longer shelf life. Liposomes-in-gel systems provide controlled drug release, ideal for skin conditions like psoriasis, ensuring prolonged drug retention on the skin surface. SLNs, due to their solid lipid core, offer sustained drug release [28]. Ethosomes provide better drug encapsulation and enhanced release compared to traditional liposomes. DTX-LP-G demonstrate superior skin penetration, especially with small-sized liposomes, which effectively traverse the stratum corneum into deeper layers of the skin, making them ideal for treating psoriasis [14]. SLNs and ethosomes, while having lower penetration compared to liposomes, can be optimized for enhanced permeability. Nanoemulsions also show good penetration, though they are less effective than liposomes-in-gel in deeper skin layers.

### 2.4. Results of Kinetic Studies of DTX-Loaded Formulations

Based on previous in vitro drug release results, the release behavior of DTX-loaded formulations under relatively optimal preparation conditions was analyzed using mathematical models, as shown in Table S1. Interestingly, it was found that both DTX and its formulations complied with the primary release formula and the Korsmeyer–Peppas formula (R^2^ > 0.9). Among them, DTX-LP and DTX-G were more consistent with the primary release model, whereas DTX solution and DTX-LP-G were more consistent with the Korsmeyer–Peppas model. The power index of time (t) in the Korsmeyer–Peppas model is less than 0.5, the release behavior can be referred to as Fickian diffusion; between 0.5 and 1, it is called non-Fickian diffusion [29]. The fitting equation for the DTX-LP-G release curve is as follows: Q = 0.0201t^0.2849^, and the power index of t is 0.2849, indicating that the release mechanism follows the Fickian diffusion law. Pharmaceutical formulations in line with the Fickian diffusion model focus on concentration gradient-driven passive diffusion, which has the advantages of sustained-release long-term effect, high stability, and controllable design, and is suitable for the treatment of diseases that require continuous dosing, such as skin diseases, chronic diseases, etc. [30].

In the skin permeation study, the release behavior of DTX-G best fits the one-stage release model (Table S2). The cumulative drug release rate of DTX-G is low, making it difficult to reach the effective therapeutic concentration of the drug [31]. The DTX solution, DTX-LP, and DTX-LP-G all best fit the Korsmeyer–Peppas mathematical model (R^2^ > 0.9). According to the results of Korsmeyer–Peppas model fitting, DTX and DTX-LP are non-Fickian diffusion [32]. Among them, DTX-LP had a low cumulative drug release rate, which could be attributed to the increased resistance caused by vesicle contraction during liposomal translocation [33]. In addition, for DTX-LP-G, Fickian diffusion is the main release mechanism to deliver the drug into the skin by diffusion and increase the penetration rate.

### 2.5. In Vitro Antioxidant Activity of DTX-Loaded Formulations

**Figure 4:**
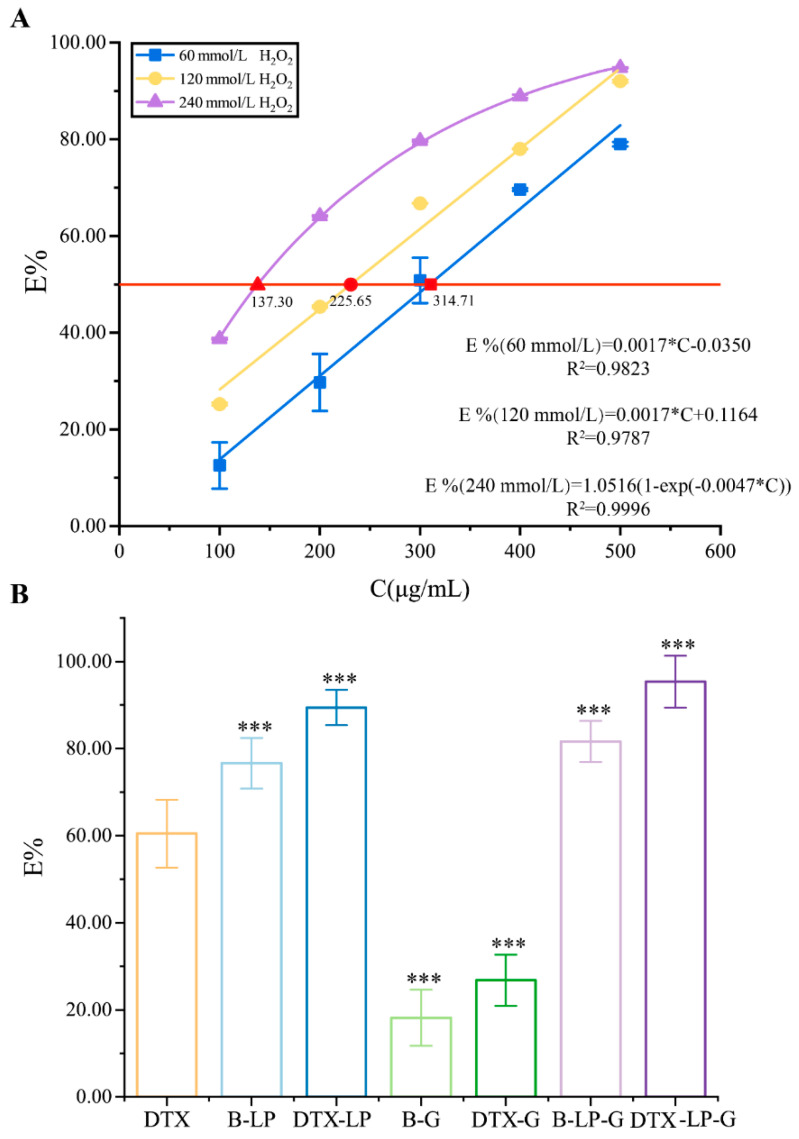
Scavenging rate of DTX, DTX-LP, DTX-G, and DTX-LP-G on H_2_O_2_. (**A**) Scavenging rates of DTX solutions for different concentrations of H_2_O_2_ (60, 120, and 240 mmol/L). The red line represents the IC_50_ value; (**B**) scavenging rate of 0.25 mg/mL DTX solution and its formulations on 120mmol/L H_2_O_2_. *** *p* < 0.001 vs. DTX.

Since H_2_O_2_ can provide hydroxyl radicals to initiate free radical chain reactions, different concentrations of hydrogen peroxide induced different degrees of oxidation [34]. Therefore, the effect of antioxidant activity of DTX was explored by setting different concentrations of the H_2_O_2_ solution (60 mmol/L, 120 mmol/L, 240 mmol/L), and the results are shown in Figure 4A. The scavenging of H_2_O_2_ by DTX increased with the increase in H_2_O_2_ concentration. The scavenging rate of H_2_O_2_ by 0.5 mg/mL DTX was close when the concentration of H_2_O_2_ solution was 120 mmol/L and 240 mmol/L, both of which reached more than 90%. As in the inflammatory microenvironment or tumor microenvironment, the increase in oxidation is to promote the release of DTX and drug efficacy [35]. In addition, when the concentration of H_2_O_2_ was 60 mmol/L or 120 mmol/L, there was a linear relationship between the clearance of H_2_O_2_ by the different concentration of DTX, and when the concentration of H_2_O_2_ was 240 mmol/L, a logarithmic relationship was formed between the clearance and the concentration of DTX, which was inhibited by the increase in the concentration of H_2_O_2_. Therefore, a concentration of 120 mmol/L H_2_O_2_ was selected for the comparison of antioxidant activity between DTX and its preparations.

At the DTX concentration of 0.25 mg/mL, both DTX and its preparations scavenged 120 mmol/L H_2_O_2_ (Figure 4B). As a whole, the H_2_O_2_ scavenging rates of DTX-LP, DTX-G, and DTX-LP-G were higher than their corresponding blank preparations. The strongest clearing effect of DTX-LP-G was observed, followed by DTX-LP, both of which were significantly more effective than DTX solution. The weakest clearing effect was found in DTX-G, probably due to the binding of DTX in the three-dimensional network structure of the hyaluronic acid hydrogel and the insensitivity to H_2_O_2_ leading to its low clearing rate. However, the scavenging effect of DTX-LP-G was higher than that of DTX-LP and DTX-G, which was also consistent with the experimental results of scavenging DPPH free radicals, suggesting that soy lecithin, cholesterol, and hyaluronic acid had the synergistic effect on scavenging H_2_O_2_, which improved the antioxidant capacity of DTX-LP-G [36].

### 2.6. Alleviation of IMQ-Induced Psoriasis by DTX-Loaded Liposomes-in-Gel

**Figure 5:**
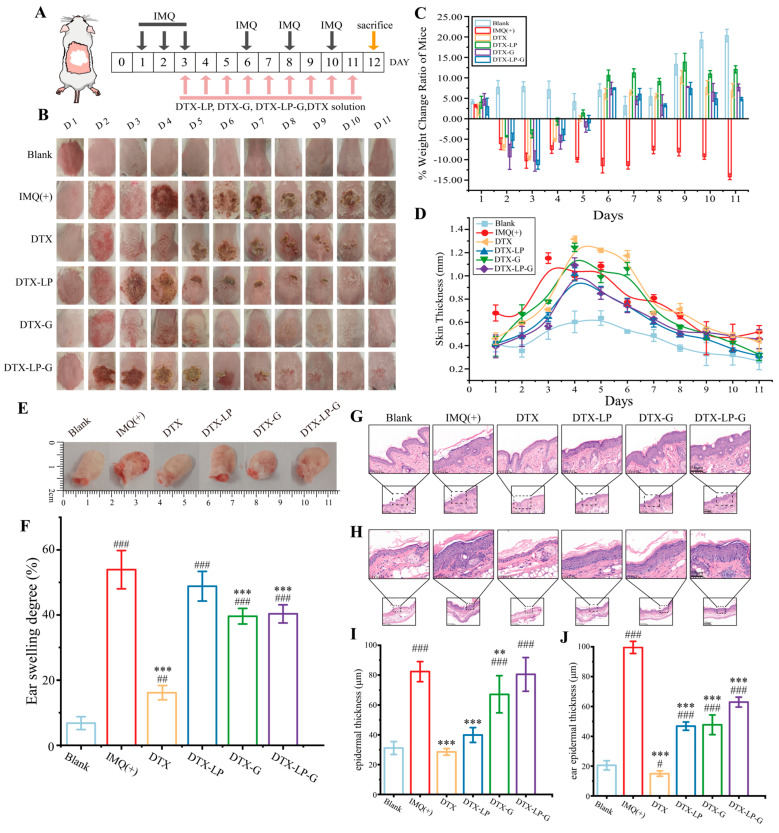
DTX-loaded formulations alleviated the psoriasis-like skin induced by IMQ. (**A**) Schematic diagram of the modeling protocol. (**B**) Phenotypic characterization of animals treated continuously for 11 days with PBS, DTX-loaded preparations (DTX-LP, DTX-G, and DTX-LP-G), and DTX solution. (**C**,**D**) Weight change ratio and skin thickness of mice during the experiment period. (**E**) Physical appearance of the right ears at the end of this study. (**F**) Ear swelling degree of mice in blank group, IMQ-induce group, and different treatment groups. (**G**) HE staining results of skin tissues in different groups. Scale bar = 100 μm. (**H**) HE staining results of ear tissues in different groups. Scale bar = 50 μm. (**I**,**J**) Epidermal thickness (**I**) and ear epidermal thickness (**J**) in different groups. # *p* < 0.05, ## *p* < 0.01, ### *p* < 0.001 vs. Blank group; ** *p* < 0.01, *** *p* < 0.001 vs. IMQ (+) group.

To confirm the therapeutic effect of DTX-LP-G, the psoriasis model was established using imiquimod cream on the dorsal skin and ears of mice and treated it with DTX-loaded preparations (Figure 5A). The experimental results indicated that DTX, DTX-LP, DTX-G, and DTX-LP-G were all effective in alleviating symptoms of psoriasis on the back skin of mice, as shown in Figure 5B. Since the beginning of the treatment, compared with the mice in the only IMQ-induced group, the mice showed a continuous increase in body weight, and the sustained decline in skin thickness, psoriasis-like area, and severity index scores (Figure 5C,D and Figure S1). Among them, the mice in positive treatment group showed negative weight gain, which might be due to the side effects of the dexamethasone as a kind of glucocorticoid drug causing body weight loss, and even slightly lower skin thickness than the normal group mice. Among them, DTX-LP was the first to restore normal psoriasis symptoms. In addition, the blood vessels in the ear were dense and the epidermal layer was thin, so its redness, swelling, and scaling was most obvious (Figure 5E). Although DTX-loaded formulations reduced ear swelling, the therapeutic effect on ear swelling was not as good as that of DTX group due to the slow and sustained release from DTX-LP, DTX-G, and DTX-LP-G (Figure 5F). In order to analyze the stratum corneum and dermis of the skin and ear tissues of the mice in each group, the tissue sections were observed by the HE staining as shown in Figure 5G,I. Compared with the blank group, mice in the IMQ group showed significantly enhanced keratinization, epidermal protrusion, capillary dilatation, congestion, and cellular infiltration, consistent with psoriasis symptoms [37]. Mice treated with DTX solution showed significant recovery of pathological changes compared to the IMQ group. The mice in the DTX-LP and DTX-G groups showed varying degrees of recovery of the skin and ears. The thickness of the stratum corneum of the mice in the DTX-LP-G group was almost unchanged, but there was a significant reduction in the thickness at the ears there (Figure 5H,J). These results were also consistent with the measurement of skin thickness using a vernier caliper.

DTX modulates the pathological process of psoriasis through a dual mechanism of action. On one hand, as a microtubule-stabilizing agent, it inhibits microtubule depolymerization and induces cell cycle arrest at the G2/M phase, thereby suppressing the abnormal proliferation of keratinocytes. On the other hand, it alleviates inflammatory responses by inhibiting T-cell activation and dendritic cell function, which reduces the release of pro-inflammatory cytokines (such as IL-17 and TNF-α) and mitigates the hyperactivation of immune cells. This dual mechanism effectively targets both the hyperproliferation and immune dysregulation underlying psoriasis pathogenesis.

### 2.7. Results of Enrichment Analysis of Docetaxel and Psoriasis

**Figure 6:**
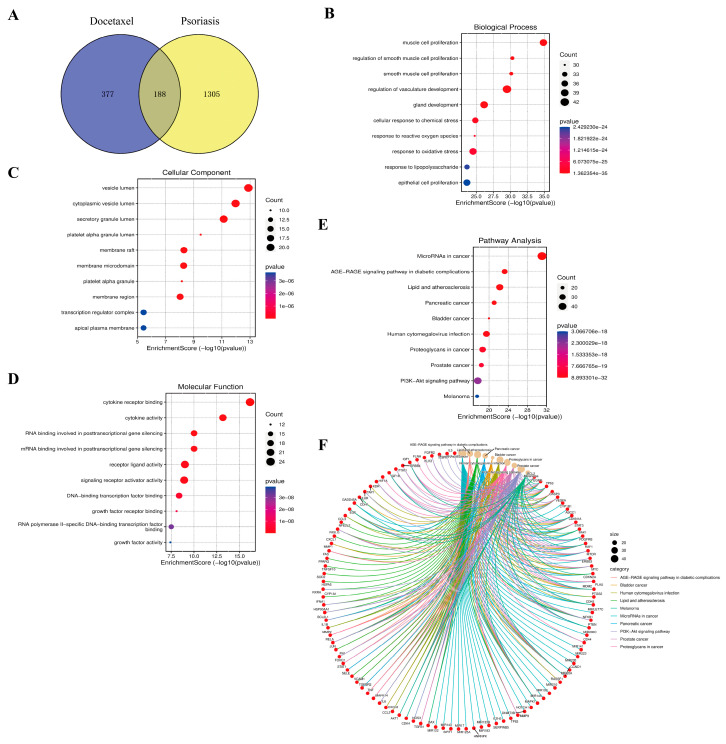
Results of the network pharmacology analysis of docetaxel and psoriasis. (**A**) Venn diagram of psoriasis and docetaxel. (**B**–**E**) Dot-plot diagrams for BP enrichment, CC enrichment, MF enrichment and KEGG pathway enrichment (Dots showed the number gene count, the x-axis showed logP and color showed the *p*-value). (**F**) Chord diagram of top 10 highly enriched pathways and equivalent targets from KEGG pathways enrichment analysis.

From the Gene Cards database, 1493 psoriasis targets and 565 docetaxel targets were collected and 188 intersecting targets were screened by applying Venn diagram (Figure 6A). The intersecting targets were then analyzed for GO and KEGG enrichment by applying the David database. The top 10 significantly enriched biological process (BP), cellular component (CC), and molecular function (MF) terms were studied. Among them, BP was primarily associated with the muscle cell proliferation, regulation of vasculature development, cellular response to chemical stress, and response to reactive oxygen species (Figure 6B). CC mainly consisted of vesicle lumen, cytoplasmic vesicle lumen, secretory granule lumen, membrane raft, membrane microdomain, and membrane region (Figure 6C). MF was chiefly involved in cytokine receptor binding, RNA binding involved in posttranscriptional gene silencing, mRNA binding involved in posttranscriptional gene silencing, and signaling receptor activator activity (Figure 6D).

KEGG enrichment analysis showed that MicroRNA in cancer, AGE-RAGE signaling pathway in diabetic complications, lipid and atherosclerosis, pancreatic cancer, bladder cancer, human cytomegalovirus infection, proteoglycans in cancer, prostate cancer, PI3K-Akt signaling pathway, and melanoma were the top 10 signaling pathways (Figure 6E). As shown in Figure 6F and Table S3, 101 key targets were associated with in the top 10 signaling pathways, e.g., Bcl-2, EGFR, IL6, VEGF, HIF-1α, etc. Among them, the AGE-RAGE signaling pathway is mainly associated with aging. Studies have shown that changes in NAD, AGE, RAGE, CRP and elastin levels within the serum of psoriasis patients are potential markers of psoriasis severity and aging [38]. In addition, the down-regulation of the AGE-RAGE pathway for anti-inflammatory purposes was predicted and experimentally validated by network pharmacology to inhibit the expression of inflammatory factors in psoriasis [39]. Next, the PI3K/Akt signaling pathway has been repeatedly reported to regulate the hyperproliferation of keratinocytes [40,41]. PI3K consists of three molecules with different functions, structures, and types of molecules, represented by class I, II, and III, which are involved in cell growth and proliferation [42]. Then, PI3K binds to Akt and controls the downstream targets of mTOR, FOXO, etc., and exerts a negative regulation of cell proliferation [43]. The PI3K/Akt pathway is also involved in various immune-mediated inflammation, activating cells to release a large number of cytokines (IL1β, IL6, IL22, IL23, CXCL, etc.). Akt is also involved in various immune-mediated inflammation pathway, activating cells to release large amounts of cytokines (IL6, IL12, IL22, CXCL, etc.) [42]. Moreover, DTX possesses the ability to block the PI3K/Akt signaling pathway, enhancing its cytotoxicity and inhibiting cell growth and proliferation [44]. In conclusion, it illustrated that DTX might treat psoriasis via modulating the AGE-RAGE pathway and PI3K/Akt signaling pathway via KEGG analysis.

### 2.8. DTX-Loaded Liposomes-in-Gel Regulating Immunity and Inhibiting MDA Production

**Figure 7:**
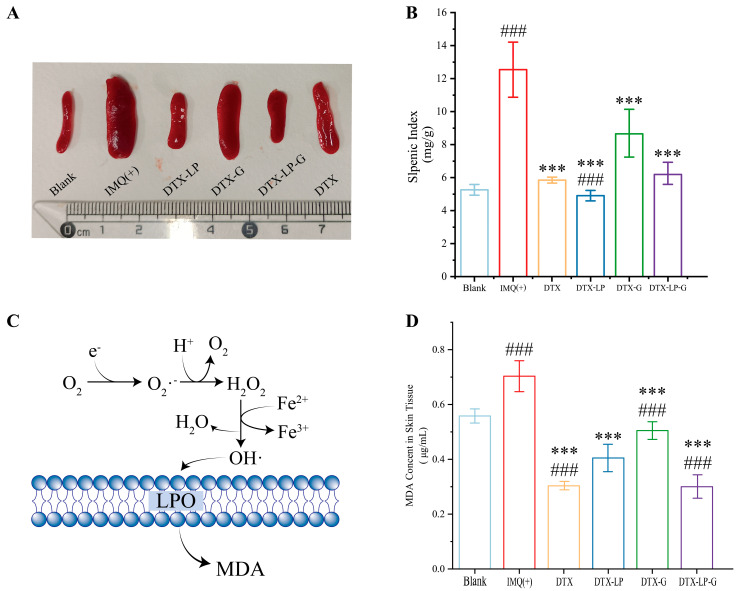
DTX-loaded formulations regulated immunity and inhibited MDA production: (**A**) the size of spleen; (**B**) splenic index (mg/g) of mice in blank group, IMQ-induce group, and different treatment groups; (**C**) hydrogen peroxide-generated free radicals catalyzed by ferrous ions, and free radicals attack lipid membranes to generate MDA; (**D**) MDA content in skin tissues of each group. ### *p* < 0.001 vs. blank group; *** *p* < 0.001 vs. IMQ (+) group.

Splenomegaly has become the sign of the overreaction of the immune system due to the spleen being the most important immune organ in the body. It had been shown that splenic tyrosine kinase inhibition altered dendritic cell function to suppress psoriasis-like inflammation in mice [45]. Therefore, the spleen status of each group was tested after treatment. As shown in Figure 7A,B, the splenic index of the model group was approximately twice that of the blank group, indicating that the immune response was activated. After treatment with DTX, DTX-LP, DTX-G, and DTX-LP-G, the size and index of the splenic decreased. Except for the DTX-G group, there was no significant difference in the splenic index among DTX, DTX-LP, DTX-LP-G, and the blank group. On the one hand, the spleen is the main site of immune cell development and growth. There are currently several explanations for the pathogenesis of psoriasis, of which T-cell activation is the most vital. T cells, as immune cells, release a variety of cytokines and chemokines that interact with keratin-forming cells and other immune cells (dendritic cells, Langerhans cells, neutrophils) to form a complex network of cytokines that mediate and sustain psoriatic inflammation [46]. On the other hand, the presence of splenomegaly in psoriasis reflects the fact that it is a systemic immune disease [47]. DTX relief of splenomegaly suggests that DTX-containing formulations transport DTX to the deeper layers of the skin and even into the blood vessels for systemic treatment, either in the form of vesicles or in a permeably released manner. Therefore, DTX and DTX formulations effectively alleviated psoriasis like skin by regulating immune function and were expected to have greater potential in future clinical applications.

Next, the mechanism of free radical generation within the cell by hydrogen peroxide was also related to the intake of excess iron ions. Cells that ingest excess iron ions trigger the Fenton reaction, causing lipid peroxidation [48]. Once lipid peroxidation occurs, lipids were first involved in the reaction. During the reaction, a large amount of reactive oxygen species (ROS) and some oxidation products appeared (Figure 7C). MDA, as a lipid peroxidation product, could bind to biological macromolecules (e.g., DNA, proteins, etc.), thus affecting the structure of biological membranes, leading to cell membrane rupture and cytotoxic reactions [49,50]. In contrast, antioxidants might react with oxidation products or oxidative enzymes, etc., directly or indirectly inhibiting MDA production and reducing cellular damage [51]. In previous reports on the pathogenesis of psoriasis, effective modulation of oxidative stress in vivo was a feasible way to treat psoriasis [8,52]. Therefore, the MDA content in skin tissues of mice of six groups was measured (Figure 7D). Compared with the model group, both DTX, DTX-LP, DTX-G, and DTX-LP-G significantly reduced the MDA content, especially DTX and DTX-LP-G. This also reflected the advantages of the liposomes-in-gel system in delivering DTX to the skin. Although DTX-LP and DTX-G also reduced the MDA content, the cumulative concentration of the drug was insufficient to achieve a therapeutic effect comparable to that of the DTX solution due to their poor penetration into the skin.

### 2.9. DTX-Loaded Liposomes-in-Gel Inhibiting Angiogenesis via Regulating IL6-HIF-1α-VEGF Axis

**Figure 8:**
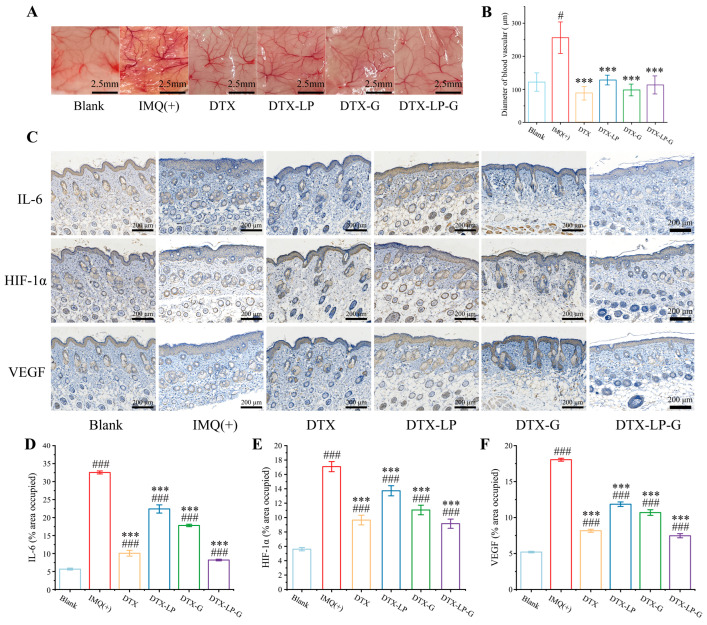
DTX-loaded formulations inhibited microvascular abnormalities and relative expression of IL6, HIF-1α, and VEGF. (**A**) Visual observation of subcutaneous blood vessels in each group. (**B**) The diameter of blood vessels in each group. (**C**) Representative images of IL6, HIF-1α, and VEGF expression in each group detected by immunohistochemistry. Scale bar = 200 μm. (**D**–**F**) The area of the region occupied by IL6, HIF-1α, and VEGF expression in each group. # *p* < 0.05, ### *p* < 0.001 vs. blank group; *** *p* < 0.001 vs. IMQ (+) group.

Microvascular abnormalities are the representative feature of psoriasis, and activated keratinocytes in lesional skin accelerate epidermal cell renewal and promote the expression of pro-angiogenic cytokines [53]. Therefore, the degree of vascular distribution and the size of vessel diameter were observed (Figure 8A,B). The IMQ-induced blood vessel distribution in the model group was dense and reached 256 ± 42.75 nm, which was almost twice the diameter of the blood vessels in the blank group and much higher than that of DTX, DTX-LP, DTX-G, and DTX-LP-G. In addition, the subcutaneous blood vessels of mice treated with DTX, DTX-LP, DTX-G, and DTX-LP-G were denser than those of the blank group, but there was no statistically significant difference in blood vessel diameters from those of the normal group, which suggested that DTX and its preparations all played a role in restoring blood vessels.

In the pathogenesis of psoriasis, a large number of inflammatory factors (TNF-α, IL1, IL6, IL17, IL22, IL23, etc.), pro-angiogenic factors (EFG, VEGF, HIF-1α, etc.) are released by dendritic cells, neutrophils, macrophages, and pathogenic T cells, which are the main cause of subcutaneous vascular abnormalities [54]. In addition, VEGF was involved in seven of the top ten pathways according to the KEGG enrichment analysis, which highlighted the importance of anti-VEGF in psoriasis treatment. Moreover, IL6 is located upstream of the HIF-1α signaling pathway and activates STAT3 by triggering a signaling cascade, which in turn promotes the expression of HIF-1α, thereby activating the VEGF signaling pathway and promoting vascular proliferation [55]. Therefore, to further investigate DTX and its agents to alleviate the development of psoriasis by modulating the expression of pro-angiogenic factors, IL6, HIF-1α, and VEGF in the dorsal skin of mice was examined by immunohistochemistry (Figure 8C). As IMQ activates Toll-like receptors, it stimulates the production of various factors such as interleukins, pro-angiogenic factors, and so on [56]. As a result, the expression area of IL6, HIF-1α, and VEGF in the IMQ-induced group was much higher than that in the blank group (Figure 8D–F). In addition, DTX and its preparation group had different degrees of inhibition on the expression levels of IL6, HIF-1α, and VEGF, especially DTX-LP-G, which also indicated that the liposomes-in-gel system had good skin permeability and therapeutic effect. In previous studies, DTX has been found to down-regulate VEGF, HIF-1α, and other related pro-angiogenic factors [57], which was likely to be the mechanism by which DTX controlled microvascular abnormalities in psoriasis. In addition, IL6, HIF-1α, and VEGF played important roles in the pathogenesis of many subtypes of psoriasis, such as pustular psoriasis [58], psoriatic arthritis [59], etc., which also suggested that DTX could treat many subtypes of psoriasis by preventing IL6 from activating the HIF-1 signaling pathway.

## 3. Conclusions

In this paper, cholesterol and soy lecithin with a weight ratio of 1:5 were used to construct a lipid membrane support, and then the liposomes-in-gel system was prepared to deliver DTX across the skin stratum corneum for the topical treatment of psoriasis in the skin. This study primarily employed an imiquimod-induced psoriasis model, which predominantly simulates plaque-type psoriasis. Therefore, DTX-LP-G demonstrates greater efficacy in treating localized plaque psoriasis. DTX-LP-G had a high drug loading capacity, moderate viscosity, and good skin permeability and stability. DTX-LP-G not only showed efficient clearance on hydrogen peroxide in vitro but also inhibited the generation of MDA from oxidative stress in skin tissues in a psoriasis model. In addition, DTX and its preparations regulated the immune system to inhibit splenomegaly, especially DTX-LP-G. In spite to this, DTX-LP-G inhibited the expression of IL6, VEGF, and HIF-1α, which suggested that DTX played the anti-inflammatory and anti-angiogenic effects. In conclusion, DTX-LP-G was suitable for transdermal delivery of DTX for the treatment of psoriasis, and the liposomes-in-gel system might provide a reference for the development of transdermal delivery formulations of drugs. The potential limitations of DTX-LP-G lie in the possible degradation or oxidation of liposomes and the drug during storage, which may reduce their activity. Therefore, it is necessary to use highly stable lipid materials (such as hydrogenated phospholipids) and antioxidants to enhance the chemical stability of the liposomes. Additionally, employing microfluidic technology or high-pressure homogenization can improve the uniformity and reproducibility of liposome preparation, thereby facilitating their clinical application and development. DTX-LP-G exhibits promising potential for the treatment of psoriatic arthritis.

## 4. Materials and Methods

### 4.1. Materials

DTX (purity 98.0%) was purchased from Hubei Huizepu Pharmaceutical Technology Co., Ltd. (Caidian, Hubei, China). Cholesterol and soy lecithin were purchased from Beijing Yuan Hua Mei Lecithin Sci-Tech Co., Ltd. (Beijing, China). Imiquimod ointment (IMQ) was purchased from Sichuan Med-shine Pharmaceutical Co., Ltd. (Chengdu, Sichuan, China). Anti-IL6 (GB11117), anti-VEGF (PB9253), anti-HIF-1α (AF5131), and remaining reagents required for immunohistochemistry were provided by AiFang Biological Co., Ltd. (Changsha, Hunan, China). Thiobarbituric acid (TBA) was purchased from Shanghai Yuanye Co., Ltd. (Shanghai, China). Hydrogen peroxide (H_2_O_2_) was provided by Shanghai SuYi Chemical Reagent Co., Ltd. (Shanghai, China). Trichloroacetic acid (TCA) was provided by Shanghai Macklin Biochemical Technology Co., Ltd. (Shanghai, China). Potassium dihydrogen phosphate was provided by Xilong Scientific Co., Ltd. (Shantou, China). All the other chemicals and solvents were of analytical reagent grade.

### 4.2. Preparation and Characterization of DTX-Loaded Liposomes

Liposomes were prepared according to the injection method described [60]. Specifically, cholesterol (CHO)/soy lecithin (SL) (1:3, 1:5, and 1:10, *w*/*w*) and 5 mg DTX were dissolved in 4.0 mL anhydrous ethanol. Under magnetic stirring, the above drug-containing mixture was added to 10.0 mL of PBS using a syringe at a certain speed. At room temperature, the liposome suspension was continuously stirred for about 2 h. Finally, the DTX-loaded liposome (DTX-LP) was filtered across the microporous membrane with a pore size of 0.22 μm, and the obtained concentration of 0.5 mg/mL was transferred into a graduated test tube for standby. According to the preparation process, no DTX in the organic phase was obtained from the blank liposomes (B-LP).

Next, DTX-LP prepared with different mass ratios of cholesterol and soy lecithin (1:3, 1:5, and 1:10) was mixed, and morphological features were observed using cryo-electron microscopy (Glacios-200KV, Thermo Fisher Scientific, Inc., Waltham, MA, USA), particle size and zeta potential were determined using a Malvern nano-particle sizer (ZEN3690, Malvern, PA, USA), and pH value was measured using a pH meter (PHSJ-4A, Shanghai Leici Instruments Co., Ltd., Shanghai China). Finally, high speed centrifugation was used to determine the encapsulation efficiency (EE%) and drug loading rate (DLR%). A total of 0.3 mL of the intercepted solution was taken and 2.7 mL of ethanol for ultrasonic demulsification was added for 30 min. The content of DTX encapsulated in liposomes was determined by UV visible spectrophotometry. Before filtration, 0.3 mL of DTX-LP was added to 2.7 mL of anhydrous ethanol for ultrasonic, and the total content of DTX in DTX-LP was measured. EE% and DLR% were calculated according to the following equation.

$$\mathrm{EE}\;(\%)=(1-\frac{M_{1}}{M_{2}})\times 100\%$$

$$\mathrm{DLR}\;\left(\%\right)=\frac{{M_{2}-M}_{1}}{M_{3}}\times 100\%$$

where “M_1_” is the mass of DTX unencapsulated; “M_2_” is the total mass of DTX measured; and “M_3_” is total mass of carrier material and drugs in DTX-LP.

### 4.3. Optimization of Hyaluronic Acid Content in DTX-Loaded Gel and DTX-Loaded Liposomes-in-Gel

According to the methods reported in previous literature, 1% hyaluronic acid (HA) is commonly used as the gel skeleton in the liposomes-in-gel delivery system [61]. Therefore, 0.5%, 1%, and 2% HA were selected as concentration of the gel matrix for loading DTX, which was added to the DTX solution and naturally solubilized overnight to obtain a 0.5 mg/mL DTX-loaded gel (DTX-G). In addition, the liposomes-in-gel system was prepared with reference to the method reported in our laboratory [62]. Briefly, HA was added to 10.0 mL of the DTX-containing liposome suspension, stirred at room temperature, and left overnight to allow complete dissolution to obtain DTX-loaded liposomes-in-gel (DTX-LP-G). Similarly, we prepared blank gel (B-G) and blank liposomes-in-gel (B-LP-G) without DTX according to the above technique. 

Then, the DLR% of DTX-G and DTX-LP-G was measured in the same determination method as that of DTX-LP. And the viscosity and pH value of DTX-G and DTX-LP-G were determined with an Uchis capillary viscometer and pH meter, respectively.

### 4.4. In Vitro Drug Release Study

The release behavior of DTX formulations was evaluated using improved dialysis membrane methods [63]. In short, the diffusion experiment was carried out at a constant temperature of 37 ± 1 °C controlled by a constant-temperature magnetic agitator. Then, 1.5 mL of sample was put into the upper compartment. Two milliliters of solution from the lower compartment of the chamber were collected at the different time of 0.5 h, 1 h, 2 h, 3 h, 4 h, 5 h, 6 h, 7 h, 8 h, 9 h, 10 h, 11 h, 12 h, 24 h, 36 h, 48 h, …, 96 h, and PBS buffer with the same volume and temperature was added. Thereafter, their absorbance was measured at 230 nm and recorded. The test was repeated three times. The percentage of the released DTX at each time point was calculated from the following equation:

$$Released\;DTX\left(\%\right)=\frac{Q_{T}}{Q_{R}}\times 100\%$$

where “Q_T_” is the cumulative amount of drug released at each time point and “Q_R_” is the total amount of drug in the formulation.

### 4.5. Skin Permeation Study

According to the in vitro drug release experiment, the dialysis membrane was replaced with bare mouse back skin, and the diffusion experiment lasted for 48 h. The remaining steps were the same. In order to further investigate the drug deposition, the mouse skin after diffusion was subjected to determine the drug content in the dorsal skin by the sonication for subsequent research.

### 4.6. Kinetic Studies

Fitting a curve involves using a specific mathematical model to derive a series of sequence curves data, to study the internal relationship between two data sets, and to understand the variation between the data. Therefore, in order to study the mechanism of drug release from each set of samples, a variety of fitting models in Origin 2019b were selected for determining the best fitting curves (Table 3).

### 4.7. Extracorporeal Clearance on H_2_O_2_

The clearance hydrogen peroxide (H_2_O_2_) experiments could be evaluated the resistance of DTX and DTX-loaded formulations to free radical attacks [64]. For the sample group, 0.6 mL of DTX solution was taken in a test tube, and 1.8 mL of H_2_O_2_ (concentrations of 60 mmol/L, 120 mmol/L, 240 mmol/L) was added, and the reaction was carried out at room temperature for 10 min. The absorbance was measured at 230 nm and recorded as A_S_. The absorbance values of the blank group (PBS solution plus different concentrations of H_2_O_2_) and the control group (the sample group concentration of DTX solution plus PBS solution) were determined simultaneously and the absorbance values were recorded as A_B_ and A_C_, respectively. The clearance of hydrogen peroxide (E%) was calculated according to the following equation. In addition, after determining the appropriate concentration of hydrogen peroxide, DTX-LP-G, DTX-LP, and DTX-G with the concentration of 0.5 mg/mL and the corresponding blank preparation were taken as samples and operated in the same way to calculate the clearance to hydrogen peroxide.

$$E\;(\%)=(1-\frac{A_{S}-A_{C}}{A_{B}})\times 100\%$$

### 4.8. Establishment of Psoriasis Model

The Kunming male mice, weighing 22 ± 2 g and aged 4 to 6 weeks, were purchased from the Animal Experimental Center of Anhui University of Chinese Medicine (Hefei, China). All purchased mice were raised in an SPF level environment for one week to adapt to the experimental environment. The experiments were approved by the Animal Ethics Committee of Anhui University of Chinese Medicine (Animal Ethics No. AHUCM-mouse-2023136).

Then, we referred to the previous literature to establish IMQ-induced psoriasis animal model [11]. The back villi of all mice were removed with hair removal cream to obtain exposed 2 × 3 cm back skin, randomly divided into groups with 5 mice in each group. Except for the blank group, the remaining five groups of mice were coated with IMQ-inducing dorsal skin as well as the right ear only for the first three days. From day 3, PBS, DTX-loaded preparations (DTX-LP, DTX-G, and DTX-LP-G), and DTX solution were applied to the same area until day 11. In addition to this, 62.5 mg IMQ was applied on days 6, 8, and 10 to maintain the psoriasis-like skin.

### 4.9. PASI Score

Psoriasis mainly manifested as erythema, scaling, and skin thickening on the skin. The skin condition of mice was objectively scored based on the Psoriasis Area and Severity Index (PASI), and we needed to perform scoring every day during the experiment. The erythema, scaling, and thickening of the skin were scored independently based on the severity of each characterization on a scale of 0–4 as follows: 0, none; 1, slight; 2, moderate; 3, marked; and 4, very marked.

### 4.10. Measurement of Ear Swelling and Splenic Index

On the 12th day of the experiment, all mice were anesthetized after being weighted. Blocks of tissue of the same size were taken from the right and left ears with a perforator and noted as W_L_ (mg) and W_R_ (mg). Next, the spleen tissue was taken out, washed clean, dried with filter paper, weighed, and recorded as W_S_ (mg). The degree of ear swelling and the splenic index (SI, *w*/*w*) were calculated according to the following formula.

$$\mathrm{Ear}\;\mathrm{swelling}\;\mathrm{degree}\;\%=\frac{{W_{R}-W}_{L}}{W_{L}}\times 100\%$$

$$\mathrm{SI}\;(\mathrm{mg}/g)=\frac{W_{s}}{W_{m}}$$

where “W_s_” is the weight of spleen tissue and “W_m_” is the weight of mouse body.

### 4.11. Measurement of Blood Vessel Diameter

After anesthesia, the skin of mice at the administration site on the back was completely separated off, and the changes in blood vessel distribution were observed visually and then processed with Image-J software (1.54f) to obtain the blood vessel diameter data of mice in each group for analysis.

### 4.12. Determination of MDA Content in Tissues

Mouse tissues such as the heart, liver, spleen, lungs, kidneys, brain, and skin were collected and cleaned with PBS to remove surface blood stains. Filter paper was used to absorb the water. After being weighted, physiological saline was added to prepare the 10% tissue homogenate. According to the previous experimental method [65], the TBA colorimetric method was used to measure the amount of MDA generated in the tissue homogenate.

### 4.13. Histological Analysis

After anesthesia, the ear and back skin of mice was taken and fixed in 4% paraformaldehyde, and paraffin sections (6 μm in thickness) were made. After hematoxylin–eosin (HE) staining, the pathological changes in the skin tissues were observed using the microscope (E100, Nikon, Tokyo, Japan), and the epidermal and ear thickness were quantified using Image J software (1.54f).

For immunohistochemical analysis, paraffin sections of skin tissue were deparaffinized and hydrated, and then the sections were treated with 3% hydrogen peroxide for 15min at room temperature. After blocking non-specific antigens, sections were incubated overnight at 4 °C with primary antibodies, including anti-IL6 antibody (1:200 dilution), anti-VEGF antibody (1:300 dilution), and anti-HIF-1α antibody (1:200 dilution). Afterwards, the sections were incubated with HRP-conjugated secondary antibody for 50min, followed by dropwise addition of diaminobenzidine (DAB) color development solution. Finally, the nuclei were restrained with hematoxylin after checking the staining results under the microscope (E100, Nikon, Tokyo, Japan). Images were captured with an imaging system (DS-U3, Nikon, Tokyo, Japan), and then Image J software was used to analyze the staining intensity of IL6, VEGF, and HIF-1α in 3 random fields of view of the skin samples.

### 4.14. Enrichment Analysis of Docetaxel and Psoriasis

First, targets with a relevance score of more than 1 were collected in GeneCards database (Human GeneCards Database, https://www.genecards.org/ URL (accessed on 18 March 2024)) using “psoriasis” and “docetaxel” as keywords, and duplicates were removed. Afterwards, the targets of docetaxel and psoriasis were then uploaded onto the Venny2.0 platform (https://bioinfogp.cnb.csic.es/tools/venny/ URL (accessed on 23 March 2024)), and the cross-targets of DTX and psoriasis were collected as prospective targets for further studies. In addition, the cross-targets were imported into the David database (database https://david.ncifcrf.gov/ URL (accessed on 8 April 2024)) for Gene Ontology (GO) and Kyoto Encyclopedia of Genes and Genomes (KEGG) enrichment analysis. Finally, a bioinformatics platform (https://www.bioinformatics.com.cn/ URL (accessed on 8 April 2024)) was used to generate bubble and chord plots for GO and KEGG enrichment analysis [66,67,68].

### 4.15. Statistical Analysis

All experiments were repeated no less than three times. Experimental data were processed and analyzed using Excel, Origin 2019, and SPSS 23.0 software. One-way ANOVA was used for comparison between multiple groups, and the LSD test was used for two-by-two comparisons. *p* < 0.05 was considered statistically different.

## Figures and Tables

**Table 1:** Optimization and characterization of DTX-LP prepared by different ratios of CHO:SL.

CHO:SL	Particle Size (nm)	Zeta (mV)	EE (%)	DLR (%)	pH
1:3	165.36 ± 2.94	−35.97 ± 2.19	72.59 ± 0.96	0.891 ± 0.017	7.42 ± 0.02
1:5	143.37 ± 2.04	−36.08 ± 1.08	88.50 ± 1.45 ***	0.734 ± 0.012 ***	7.40 ± 0.01
1:10	210.60 ± 3.34 *	−39.00 ± 1.26	69.17 ± 1.25 *	0.312 ± 0.009 ***	7.43 ± 0.01

The data were means ± SD (n = 3). * *p* < 0.05, *** *p* < 0.001 vs. CHO:SL (1:3).

**Table 2:** Optimization and characterization of different HA content in DTX-G and DTX-LP-G.

	HA %	Appearance	DLR (%)	Viscosity (mpa·s)	pH
DTX-G	0.5%	white	0.97 ± 0.03	5.57 ± 0.14	7.26 ± 0.01
	1.0%	white	2.21 ± 0.08 ***	7.41 ± 0.30 *	7.26 ± 0.02
	2.0%	white	1.19 ± 0.10 **	11.73 ± 1.56 ***	7.27 ± 0.02
DTX-LP-G	0.5%	milky white	0.68 ± 0.03 ***	14.28 ± 1.32 ***	7.39 ± 0.02 ***
	1.0%	light yellow	0.83 ± 0.01 *	18.72 ± 0.55 ***	7.42 ± 0.02 ***
	2.0%	light yellow	0.73 ± 0.02 **	20.22 ± 0.50 ***	7.43 ± 0.03 ***

The data were means ± SD (n = 3). * *p* < 0.05, ** *p* < 0.01, *** *p* < 0.001 vs. DTX-G (0.5%HA).

**Table 3:** Mathematical models and formulas.

Mathematical Models	Formulas
Zero order	Q=a+b×t
First order	$Q=a\times (1-e^{-b\times t})$
Higuchi	$Q=a\times (t^{\frac{1}{2}})+b$
Korsmeyer–Peppas	$Q=a\times t^{n}$

## Data Availability

Data will be made available on request.

## Supplementary Materials

The following supporting information can be downloaded at: https://www.mdpi.com/article/10.3390/gels11040228/s1, Table S1: The diffusion curve pattern of DTX solution, DTX-LP, DTX-G and DTX-LP-G; Table S2: Results of various mathematical models fitted to the ex vivo transdermal release curves of DTX and its formulations; Table S3: Top 10 highly enriched pathways from KEGG pathways enrichment analysis with ID, *p*-value, genes involved, gene count; Figure S1: Psoriasis skin score. (A) Erythema score. (B) Scaling score. (C) Skin thickening score. (D) PASI total score.
