# Supplementary material for: Liposomes-in-Gel as the Docetaxel Delivery for the Effective Treatment of Psoriasis by Inhibiting the Proliferation of Blood Vessels
Source: Gels. 2025 Mar 22;11(4):228. doi: 10.3390/gels11040228 (PMC12027167; doi:10.3390/gels11040228)

ASSOCIATED CONTENT

Supporting Information.

Table S1. The diffusion curve pattern of DTX solution, DTX-LP, DTX-G and DTX-LP-G.

	**Mathematical model**	**Equation**	**R^2**
DTX	Zero-order	$Q = 6.12 \times 10^{-5} t + 0.1593$	0.7126
	First-order	$Q = 0.3891(1 - exp(-0.0022t)$	0.9373
	Higuchi	$Q = 0.054t^{0.5} + 0.0845$	0.8896
	Korsmeyer-Peppas	$Q = 0.0349t^{0.2986}$	**0.9574**
DTX-LP	Zero-order	$Q = 4.16 \times 10^{-5} t + 0.1152$	0.6372
	First-order	$Q = 0.2803(1 - exp(-0.0020t)$	**0.9590**
	Higuchi	$Q = 0.0037t^{0.5} + 0.0619$	0.8346
	Korsmeyer-Peppas	$Q = 0.0256t^{0.2937}$	0.9077
DTX-G	Zero-order	$Q = 3.73 \times 10^{-5} t + 0.0535$	0.763
	First-order	$Q = 0.2104(1 - exp(-0.0012t)$	**0.9908**
	Higuchi	$Q = 0.0032t^{0.5} + 0.0092$	0.925
	Korsmeyer-Peppas	$Q = 0.0066t^{0.4160}$	0.9423
DTX-LP-G	Zero-order	$Q = 3.20 \times 10^{-5} t + 0.0900$	0.6996
	First-order	$Q = 0.2072(1 - exp(-0.0024t)$	0.9116
	Higuchi	$Q = 0.0028t^{0.5} + 0.0503$	0.8759
	Korsmeyer-Peppas	$Q = 0.0201t^{0.2849}$	**0.9523**

Table S2. Results of various mathematical models fitted to the ex vivo transdermal release curves of DTX and its formulations

	**Mathematical model**	**Equation**	**R^2**
DTX	Zero-order	Q=0.0721t+1.6998	0.9289
	First-order	Q=150.9226(1-exp(-6.18×E-4t)	0.9188
	Higuchi	Q=0.7424t0.5-17.7326	0.9401
	Korsmeyer-Peppas	Q=0.2333t0.8302	**0.9511**
DTX-LP	Zero-order	Q=0.0040t-0.2056	0.8674
	First-order	-	-
	Higuchi	Q=0.2117t0.5-2.1553	0.8946
	Korsmeyer-Peppas	Q=0.0035t0.9140	**0.9648**
DTX-G	Zero-order	-	-
	First-order	Q=7.1971(1-exp(-0.0217t)	**0.8722**
	Higuchi	Q=0.0724t0.5+4.5543	0.1630
	Korsmeyer-Peppas	Q=2.7307t0.1414	0.4022
DTX-LP-G	Zero-order	Q=0.0325t+12.8280	0.8031
	First-order	Q=62.5692(1-exp(-0.0019t)	0.8874
	Higuchi	Q=1.2009t0.5+7.7513	0.9824
	Korsmeyer-Peppas	Q=3.5574t0.3724	**0.9889**

The "-" means that the model failed to fit, with no equation and no R^2 value.

Table S3. Top 10 highly enriched pathways from KEGG pathways enrichment analysis with ID, P-value, genes involved, gene count.

Pathway ID	Pathway name	p-value	Gene IDs	Count
hsa05206	MicroRNAs in cancer	8.89E-32	BCL2/EGFR/TP53/CASP3/VEGFA/CYP1B1/ABCC1/CDKN1A/STAT3/ BAK1/PDGFRB/RAF1/MTOR/ERBB3/MYC/CDKN2A/PLAU/MDM2/ PTGS2/CDK6/MIRLET7C/NFKB1/PTEN/MIR200C/CD44/MIR141/MIR223/ MIR205/MIR20A/CCND1/RASSF1/MIR210/MIR100/MIR145/MAPK1/ NOTCH1/DNMT3B/MMP9/TP63/EZH2/SERPINB5/MIR133B/MIR183/ HNRNPK/MIR125A/MIR17/SIRT1/MIR143/MIR122	49
hsa04933	AGE-RAGE signaling pathway in diabetic complications	5.57E-24	BCL2/CASP3/BAX/VEGFA/STAT3/TGFB1/NOS3/CDK4/AKT1/CCL2/ NFKB1/CXCL8/IL6/MAPK14/TNF/CCND1/TGFBR2/VCAM1/SELE/MAPK1/ STAT1/FOXO1/FN1/JUN/RELA/MMP2/IL1B	27
hsa05417	Lipid and atherosclerosis	5.84E-23	BCL2/TP53/CASP3/BAX/BCL2L1/STAT3/HSP90AA1/IFNA1/CYP1A1/ RXRA/NOS3/AKT1/CCL2/NFKB1/CXCL8/IL6/MAPK14/TNF/HSPA5/ SOD2/VCAM1/SELE/TNFSF10/MAPK1/PPARG/FAS/MMP9/MMP1/JUN/ CXCL1/RELA/FASLG/NFE2L2/IL1B/CCL3	35
hsa05212	Pancreatic cancer	8.48E-22	EGFR/TP53/BAX/VEGFA/BCL2L1/CDKN1A/STAT3/BAK1/RAF1/ MTOR/TGFB1/CDKN2A/CDK4/CDK6/AKT1/EGF/NFKB1/CCND1/ TGFBR2/MAPK1/STAT1/GADD45A/RELA	23
hsa05219	Bladder cancer	1.05E-20	EGFR/TP53/VEGFA/CDKN1A/CDH1/RAF1/MYC/CDKN2A/MDM2/ CDK4/EGF/CXCL8/CCND1/RASSF1/MAPK1/MMP9/MMP1/MMP2	18
hsa05163	Human cytomegalovirus infection	3.46E-20	EGFR/TP53/CASP3/BAX/VEGFA/CDKN1A/STAT3/BAK1/IFNA1/RAF1/ MTOR/MYC/CDKN2A/MDM2/CDK4/PTGS2/CDK6/AKT1/CCL2/ NFKB1/CXCL8/IL6/MAPK14/TNF/CCND1/MAPK1/FAS/RELA/ FASLG/IL6R/TAP1/IL1B/CCL3	33
hsa05205	Proteoglycans in cancer	2.2E-19	EGFR/TP53/CASP3/VEGFA/KDR/CDKN1A/STAT3/HIF1A/IGF1R/ RAF1/MTOR/ERBB3/MYC/TGFB1/PLAU/MDM2/AKT1/CD44/ MAPK14/TNF/CCND1/MAPK1/FAS/MMP9/ITGB1/ERBB4/FN1/ IGF1/MMP2/FASLG/FLNA	31

hsa05215	Prostate cancer	3.96E-19	KLK3/BCL2/EGFR/TP53/CDKN1A/PDGFRB/HSP90AA1/IGF1R/RAF1/MTOR/PLAU/MDM2/AKT1/EGF/NFKB1/PTEN/CCND1/MAPK1/FGFR2/MMP9/FOXO1/IGF1/RELA	23
hsa04151	PI3K-Akt signaling pathway	2.24E-18	CSF3/BCL2/EGFR/TP53/VEGFA/BCL2L1/IL3/KDR/CDKN1A/PDGFRB/HSP90AA1/IFNA1/IGF1R/RAF1/MTOR/ERBB3/MYC/RXRA/NOS3/MDM2/CDK4/CDK6/AKT1/EGF/NFKB1/PTEN/IL6/CCND1/MAPK1/FGFR2/ITGB1/ERBB4/FN1/IGF1/RELA/FASLG/IL6R/CSF1	38
hsa05218	Melanoma	3.07E-18	EGFR/TP53/BAX/CDKN1A/BAK1/PDGFRB/CDH1/IGF1R/RAF1/CDKN2A/MDM2/CDK4/CDK6/AKT1/EGF/PTEN/CCND1/MAPK1/GADD45A/IGF1	20

Figure S1. Psoriasis skin score. (A) Erythema score. (B) Scaling score. (C) Skin thickening score. (D) PASI total score.

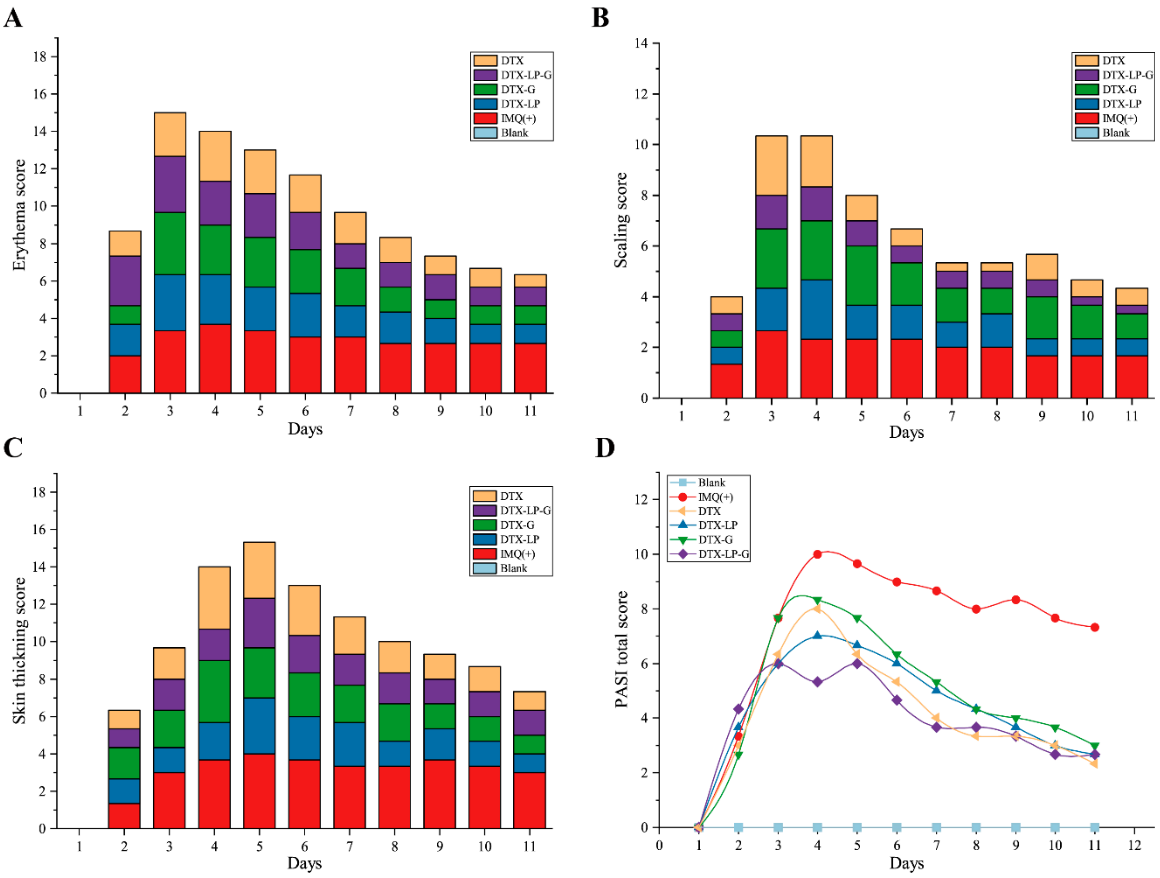

Supplement: Supplementary file 1 [file gels-11-00228-s001.zip › gels-3519050-Supplementary file.pdf]
